# pH-Dependence of Glucose-Dependent Activity of Beta Cell Networks in Acute Mouse Pancreatic Tissue Slice

**DOI:** 10.3389/fendo.2022.916688

**Published:** 2022-06-28

**Authors:** Sandra Postić, Marko Gosak, Wen-Hao Tsai, Johannes Pfabe, Srdjan Sarikas, Andraž Stožer, Dean Korošak, Shi-Bing Yang, Marjan Slak Rupnik

**Affiliations:** ^1^Center for Physiology and Pharmacology, Medical University of Vienna, Vienna, Austria; ^2^Institute of Physiology, Faculty of Medicine, University of Maribor, Maribor, Slovenia; ^3^Faculty of Natural Sciences and Mathematics, University of Maribor, Maribor, Slovenia; ^4^Taiwan International Graduate Program in Molecular Medicine, National Yang Ming Chiao Tung University and Academia Sinica, Taipei, Taiwan; ^5^Institute of Biomedical Sciences, Academia Sinica, Taipei, Taiwan; ^6^Alma Mater Europaea – European Center Maribor, Maribor, Slovenia

**Keywords:** insulin secretion, membrane excitability, potassium channels, beta cell network, collective activity, calcium waves, pancreatic islets, pH-dependence

## Abstract

Extracellular pH has the potential to affect various aspects of the pancreatic beta cell function. To explain this effect, a number of mechanisms was proposed involving both extracellular and intracellular targets and pathways. Here, we focus on reassessing the influence of extracellular pH on glucose-dependent beta cell activation and collective activity in physiological conditions. To this end we employed mouse pancreatic tissue slices to perform high-temporally resolved functional imaging of cytosolic Ca^2+^ oscillations. We investigated the effect of either physiological H^+^ excess or depletion on the activation properties as well as on the collective activity of beta cell in an islet. Our results indicate that lowered pH invokes activation of a subset of beta cells in substimulatory glucose concentrations, enhances the average activity of beta cells, and alters the beta cell network properties in an islet. The enhanced average activity of beta cells was determined indirectly utilizing cytosolic Ca^2+^ imaging, while direct measuring of insulin secretion confirmed that this enhanced activity is accompanied by a higher insulin release. Furthermore, reduced functional connectivity and higher functional segregation at lower pH, both signs of a reduced intercellular communication, do not necessary result in an impaired insulin release.

## Introduction

Extracellular and intracellular [H^+^] is typically in a nanomolar range, which is significantly lower than the concentrations of most other ions, small organic molecules, or even macromolecules like proteins and lipids. Isolated cells in culture can acutely tolerate a wider range of extracellular pH changes (pH 5-11). Cells in intact tissues are more sensitive to pH deviations and the pH of the arterial blood is tightly regulated and maintained in a very narrow range between 7.36 and 7.44. Larger local pH deviations can transiently occur in the interstitial fluids and venous blood; however, most cells cannot tolerate long-term deviations larger than half a pH unit. Also in pancreas, bicarbonate producing ductal cells could serve as a significant source of H^+^, modulating insulin release in the process of digestion and postprandial nutrient processing. Long-term pH changes are often associated with pathophysiological conditions since certain diseases, including diabetes mellitus, result in chronically lower interstitial pH (1). Interestingly manipulations of both intracellular and extracellular [H^+^] have been proposed as a potential therapeutic approach to correct the secretory defects and insulin resistance in type-2 diabetes mellitus (1, 2).

The first assessments of the effect of pH changes on the beta cell activity have been performed more than half a century ago (3). Initially, it was proposed that the observed increase in beta cell excitability was a consequence of a direct effect of low pH to reduce the membrane K^+^ permeability (Henquin, 1981). In the following years, however, the evidence has accumulated that extracellular pH primarily modulates the intracellular pH dynamics *via* regulating the Na^+^/H^+^ and HCO_3_^-^/Cl^-^ antiporters on the plasma membrane (4, 5). Intracellular pH changes were described to subsequently influence a number of processes, including glucose handling, homeostasis of cytosolic Ca^2+^ ([Ca^2+^]_c_) and exocytotic insulin release (6), and contributed to the time-dependent potentiation of insulin release (2, 6). These early results showed that the effect of the reduced extracellular pH depends strongly on the level of glucose stimulation used. At the threshold extracellular glucose concentration (6.7 mM), insulin secretion was increased at lower extracellular pH, which could be explained with increased excitability due to lower K^+^ permeability. On the other hand, at supraphysiological glucose concentrations, the effects of lowered pH were either absent (11.1 mM glucose) or even inhibitory (27.8 mM glucose) (6). To date these apparent inconsistencies have not been resolved.

Despite the fact that the described physiological effects of H^+^ load have been mostly ascribed to changes in the intracellular pH, pancreatic beta cells express several ion channels that can directly sense extracellular pH dynamics, and their direct modulation could influence the beta cell excitability as well. Two H^+^-sensitive two-pore-domain K^+^ channels, TASK1 and TALK1, have been recently shown to contribute to the polarization of the beta cell membrane potentials and thus suggesting their role in beta cell excitability and insulin release (7, 8).The importance of TALK1 channel is further emphasized by the abundancy of its transcripts in beta cells in both mice and humans (9, 10). The KCNK16 mutation causing TALK-1 gain of function results in the inhibition of glucose-stimulated membrane potential depolarization and reduced endoplasmic reticulum Ca^2+^ storage, leading to a form of maturity-onset diabetes of the young (11). In addition to the pH dependent modulation of the aforementioned K^+^ channels, other pH sensitive mechanisms have been described. Ovarian cancer G protein-coupled receptor 1 (OGR1) was described as an insulin secretion enhancer under acidic conditions through intracellular Ca^2+^ mobilization (12, 13).

We, along with other groups, have demonstrated ample communication within a collective of beta cells in a pancreatic islet. Both direct, short-range interactions through gap junctions and paracrine long-range signaling have been reported (14, 15). This intercellular communication was captured in several classical deterministic and recent stochastic models (16) reporting its role in the collective sensing and functional response of the islets. Furthermore, collective response of beta cells to glucose is disturbed in the islets from diabetic organisms (17).

The main aim of this study was to assess the effects of changes in extracellular pH on glucose-dependent beta cell collective activity in mouse pancreatic islet within the native environment of the acute pancreatic tissue slice. Our results show that the collective beta cell activation, activity, and network properties could be directly influenced by the local or global changes in the extracellular pH.

## Materials and Methods

### Ethics Statement

The study was conducted in strict accordance with all national and European recommendations pertaining to care and work with experimental animals, and all efforts were made to minimize the suffering of animals. The protocol was approved by the Federal Ministry of Education, Science and Research of the Republic of Austria (permit number: 2020-0.258.669) and by the Veterinary Administration of the Republic of Slovenia (permit number: U34401-35/2018-2).

### Tissue Slice Preparation and Dye Loading

10-30 week old outbred NMRI mice of either sex were kept on a 12:12 hours light: dark schedule in individually ventilated cages (Allentown LLC, USA) and used for the preparation of acute pancreatic tissue slices, as described previously (18–20). In brief, after CO_2_ euthanasia of mice, we accessed the abdominal cavity *via* laparotomy and injected 1.9% low-melting-point agarose (Lonza, USA) dissolved in extracellular solution (ECS, consisting of (in mM) 125 NaCl, 26 NaHCO_3_, 6 glucose, 6 lactic acid, 3 myo-inositol, 2.5 KCl, 2 Na-pyruvate, 2 CaCl_2_, 1.25 NaH_2_PO_4_, 1 MgCl_2_, 0.25 ascorbic acid) at 40°C into the distally clamped proximal common bile duct. Immediately after injection, we cooled the agarose infused pancreas with ice-cold ECS. After organ extraction, we prepared tissue slices with a thickness of 140 µm using a vibratome (VT 1000 S, Leica Microsystems, Germany) and collected them in HEPES-buffered ECS (HB-ECS, consisting of (in mM) 125 NaCl, 10 NaHCO_3_, 10 HEPES, 6 glucose, 6 lactic acid, 3 myo-inositol, 2.5 KCl, 2 Na-pyruvate, 2 CaCl_2_, 1.25 NaH_2_PO_4_, 1 MgCl_2_, 0.25 ascorbic acid; titrated to pH=7.4 using 1 M NaOH). To introduce the fluorescent Ca^2+^ indicator, we incubated the slices for 60 minutes at RT in HB-ECS (6 µM Calbryte 520-AM (AAT Bioquest, Interchim, Montluçon, France), 0.03% Pluronic F-127 (w/v), and 0.12% dimethylsulphoxide (v/v)). All chemicals were obtained from Sigma-Aldrich (St. Louis, Missouri, USA).

### Stimulation Protocol and Calcium Imaging

**Figure 1:**
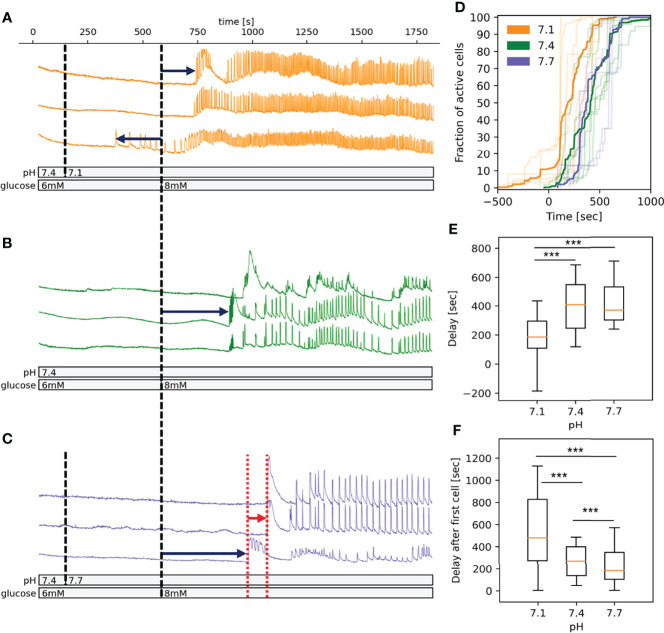
pH-dependency of activation of beta cells in islets in fresh pancreatic slices. **(A)** Time course of the [Ca^2+^]_c_ oscillations in a selection of beta cells exposed to pH 7.1 for 10 min followed by stimulatory glucose concentration (8 mM). Dark blue arrows indicate the delay time from introduction of stimulatory glucose to activation and red arrow indicates the time of delays from activation of first cell to the activation of every other cell. **(B)** As panel A at pH 7.4 **(C)** As panel A at pH 7.7. **(D)** Pooled data showing the fraction of active cells in time. The acidification induces a left shift of activation curve. Semi-transparent lines show results of the individual experiments, bold lines are the averages for each experimental condition. **(E)** Quantification of pooled data of delays from introduction of stimulatory glucose to activation. **(F)** Quantification of pooled data of delays from activation of first cell to the activation of every other cell. n=5 islets for each condition. Box plots are showing upper and lower quartile; line median; whiskers 5-95%. ***p < 0.001.

**Figure 2:**
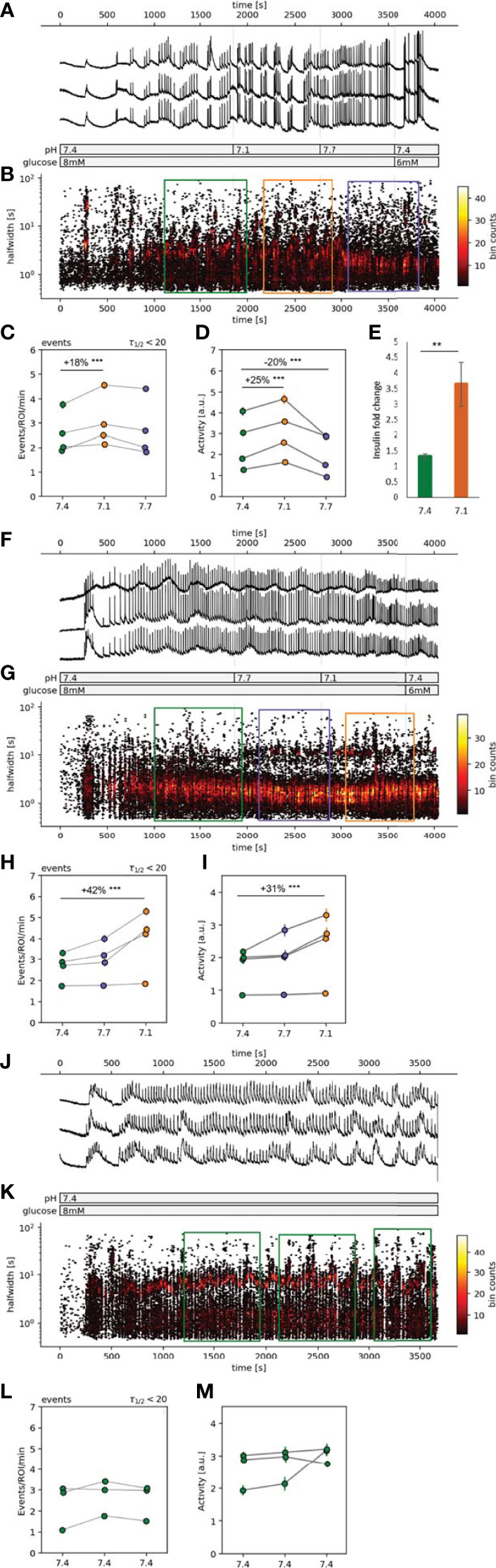
| Spatiotemporal characterization of beta cell plateau activity. **(A, F, J)** Time course of the [Ca^2+^]_c_ oscillations, exposed to 8 mM glucose stimulation protocol with pH manipulations during the stable plateau phase. **(B, G, K)** Hexbin plot of all detected [Ca^2+^]_c_ oscillations presented as the time of the peak vs. the halfwidth of [Ca^2+^]_c_ oscillation duration. The sections in the color-coded rectangles were used for the further statistical analysis (orange=pH 7.1, green=pH 7.4 and purple=pH 7.7). **(C, H, L)** Quantification of frequency of the [Ca^2+^]_c_ oscillations expressed as number of events per minute per ROI. **(D, I, M)** Quantification of the [Ca^2+^]_c_ oscillations halfwidths. **(E)** *In vitro* insulin secretion from 50 isolated islets monitored over 75 min. Data is normalized to the basal insulin secretion at 6 mM glucose (5 min incubation), and presented as mean, the error bars represent standard deviation of the mean. Experiments from the individual islets are connected with a line. (**p < 0.01, ***p < 0.001).

Individual tissue slice was transferred to an imaging chamber equipped with a perfusion system containing HB-ECS at 34°C and exposed to a square glucose stimulation pulse (8 mM) lasting for several tens of minutes. To study islet activation the pH of the HB-ECS was set to 7.4, 7.1 or 7.7 and islets were placed in a non-stimulatory glucose concentration (6 mM) at one of the pH levels, following stimulation with 8 mM glucose solution of the same pH (**Figure 1**). To study the pH-dependence of the plateau phase of the beta cell activity as well as the beta cell network properties, the pH of the extracellular solution was switched in the order 7.4 - 7.1 - 7.7 (sequence 1) or 7.4 - 7.7 - 7.1 (sequence 2) during the 1-hour exposure to stimulatory glucose of 8 mM (**Figure 2**). In the control experiments pH 7.4 was maintained throughout the duration of the experiment (**Figures 2J–L**). The imaging was performed on a Leica TCS SP5 upright confocal system (20x HCX APO L water immersion objective, NA 1.0). The acquisition frequency was set to 2 Hz and the resolution to 512x512. Calbryte 520 was excited by an argon 488 nm laser line and emitted light was detected by Leica HyD hybrid detector in the range of 500-700 nm (all from Leica Microsystems, Germany), as described previously (19).

### Glucose-Stimulated Insulin Secretion *In Vitro*

For acute insulin release in response to glucose, primary BL6J mouse islets were isolated (21) and pre-incubated (1 h) in HB-ESC buffer containing 6 mM glucose and pH 7.4 at 37°C. Islets were then incubated in HB-ESC with 6 mM glucose for 5 min (basal) after which the solution was collected and replaced with stimulatory glucose in HB-ESC 8 mM glucose with pH set to 7.4 or 7.1. Solution was collected and replaced sequentially after 5, 10 and 20 min of incubation. Insulin was determined using Insulin Ultra-Sensitive assay kit from Cisbio (Bagnols-sur-Ceze, France). Secreted insulin was normalized to the basal insulin secretion at 6 mM glucose after 5 min and shown as a fold change.

### Data Analysis

Analysis was conducted as described previously (22). In brief, we followed the analysis pipeline to first automatically detect regions of interest (ROIs) and sampled information about the time profile of [Ca^2+^]_c_ changes, their spatial coordinates, and neighboring ROIs, as well as about movie statistics, recording frequency, and pixel size. In the next step we distilled all the significant changes (events with z-score > 4) of [Ca^2+^]_c_ at all realistic time scales within each ROI. The events were characterized by the start time, their maximal height, and the width at half of their peak amplitude. The activity was calculated as a sum of all areas under the curve (AUC) of all the events detected per active ROI (ROI with more than 10 events detected), per minute. A potential non-negative nature of some of the variables (AUC, halfwidth, event rate) has been accounted for as we performed the analyses on log-transformed values.

The activation times, the start of [Ca^2+^] increases after switching from basal to stimulatory concentration, were selected manually (23). Statistics were calculated using SigmaPlot version 14 (SSPS Software). Statistical differences between groups were tested using ANOVA on Ranks followed by posthoc Dunn’s method, and the difference between two groups was tested using the Mann-Whitney U test or Student’s t-test depending on the normality of the data distribution. A more elaborate statistics, namely a mixed effects linear regression was performed where we were not only interested in the p-value but also the numerical relationship between the variables (**Figures 2C, H, L**).

### Network Analysis

To quantify the collective activity of the beta cell population in each islet we constructed functional connectivity networks, such that nodes represented individual beta cells within the tissue slices and connections between them were created based on the temporal similarity of the measured [Ca^2+^]_c_ dynamics. Specifically, two cells were considered functionally connected if their activity profiles exceeded a preset degree of coordinated activity, as described elsewhere (Pearson correlation, r>0.8)

(24–27). The resulting functional networks were diagnosed with conventional network metrics. More specifically, we assessed the coherence of beta cell activity through the average node degree, whereby a higher average node degree indicates a better aligned and more coherent [Ca^2+^]_c_ dynamics. Moreover, to quantify the degree of functional segregation, we computed the average clustering coefficient. This metrics reflects the level of clique-like structures within cell assemblies. Intuitively, a clique in a functional beta cell network represents a cohesive group of well synchronized cells, or members of a clique are more connected between each other, than to nodes outside their clique. The clustering coefficient can serve as an indicator how well particular adjacent regions within the islets are interconnected. A high value of the clustering coefficient implies a high local efficiency of information transfer as well as a good resilience to node dysfunction. For further details, see (27, 28).

To assess the nature of intercellular Ca^2+^ waves, we extracted first the fast component of [Ca^2+^]_c_ oscillations with a digital band-pass filter (cut-off frequencies 0.04 and 2 Hz). Afterwards, the traces were binarized so that the values from the onset to the end of individual oscillations were 1, and values between the oscillations were 0. The binarized signals were then used to extract individual Ca^2+^ waves by means of a space-time cluster algorithm, as described previously (29, 30). In brief, the physical positions of individual cells and their binary traces were translated to a time-space cube and all the cells that were simultaneously active within that cube were considered to belong to the same wave. In other words, we traced the course of the intercellular wave and if proximate cells became activated within a short time period, the given activation sequence was considered as one Ca^2+^ wave with a given size. Accordingly, the size encompasses the information about the number of cells involved and the duration of the wave. To compare wave sizes from different islets quantitatively, we normalized the detected wave sizes with the number of cells in the given islet.

## Results

Beta cell network within an islet was activated using a square pulse elevation from a substimulatory concentration of 6 mM to a glucose concentration above the physiological stimulatory threshold in the *in vivo* conditions for the NMRI mice (8 mM). A pH-dependence of the response of beta cell network to the stimulus protocol, which consisted of two subsequent phases, an activation and a plateau phase (**Figures 1**, **2**), was assessed and analyzed (22, 31) by either doubling (pH 7.1 = 80 nM) the physiological extracellular [H^+^] of pH 7.4 (40 nM) or halving it (pH 7.7 = 20 nM).

### pH-Dependence of Glucose-Dependent Beta Cell Activation

The spatiotemporal activation properties of individual beta cell in an optical section of an islet were examined following stepwise glucose increases at either pH 7.1, 7.4 or 7.7 (**Figures 1A–C**, respectively). Beta cells responded to the increased glucose concentration with a characteristic delay in the onset of [Ca^2+^]_c_ increase (activation delay), which at 8 mM glucose and pH 7.4 as well as at pH 7.7, lasts for about 400 s (**Figure 1**). When slices were exposed to pH 7.1 this delay shortened significantly, moreover, we observed a premature activation of ~10% of beta cells in an islet already at 6 mM glucose (**Figures 1A, D, E**), left-shifting the overall time-dependence of beta cell activation. The median beta cell activation delay at 7.1 was around 200 s (**Figure 1D**). The delay after first cell activation varied significantly between the three conditions and was the longest and most variable in pH 7.1 due to earlier activation of small clusters of beta cells (**Figures 1C, F**). As can be appreciated in **Figure 1D**, the overall activation of beta cells during stimulation had a staircase-like appearance, suggesting that clusters of beta cells rather than single cell activated at a certain time.

### pH Influences the Frequency of the [Ca^2+^]_c_ Oscillations and Average Beta Cell Activity in an Islet

The response of an islet to 8 mM glucose exhibited a biphasic response, consisting of an initial transient, asynchronous [Ca^2+^]_c_ oscillations, followed by a synchronous plateau phase with relatively regular [Ca^2+^]_c_ oscillations, presented as clearly distinguishable events (**Figures 2A, F, J**). Once the responsiveness of the beta cells in the control pH was established and a stable plateau [Ca^2+^]_c_ oscillations was observed, the pH conditions were manipulated. To assess the possible dominant and long-lasting effects of a certain pH condition we tested the pH in either sequence 1 or sequence 2 (**Figures 2B, G, K**). Elevating [H^+^] in either sequence (**Figures 2B, C, G, H**, orange circles) increased the frequency of [Ca^2+^]_c_ oscillations. The increase in oscillation frequency at lower pH was significant in comparison to control pH (**Figure 2**, green circles), 18% in sequence 1 and 42% in sequence 2, while at pH 7.7 no significant changes in oscillation frequency was observed (**Figure 2**, blue circles). The average activity of beta cells in an islet, during sequence 1 increased in pH 7.1 by 25% and in pH 7.7 it decreased by 20% (**Figure 2D**). During the sequence 2 the change in average islet activity did not change at pH 7.7 and at pH 7.1 it increased by 31% (**Figure 2I**). In control experiments, constant pH at 7.4, indicated that prolonged time of activity did not influence neither the frequency of [Ca^2+^]_c_ oscillations nor the average islet activity (**Figures 2K–M**).

### Functional Beta Cell Networks, Ca2+ Waves and pH

**Figure 3:**
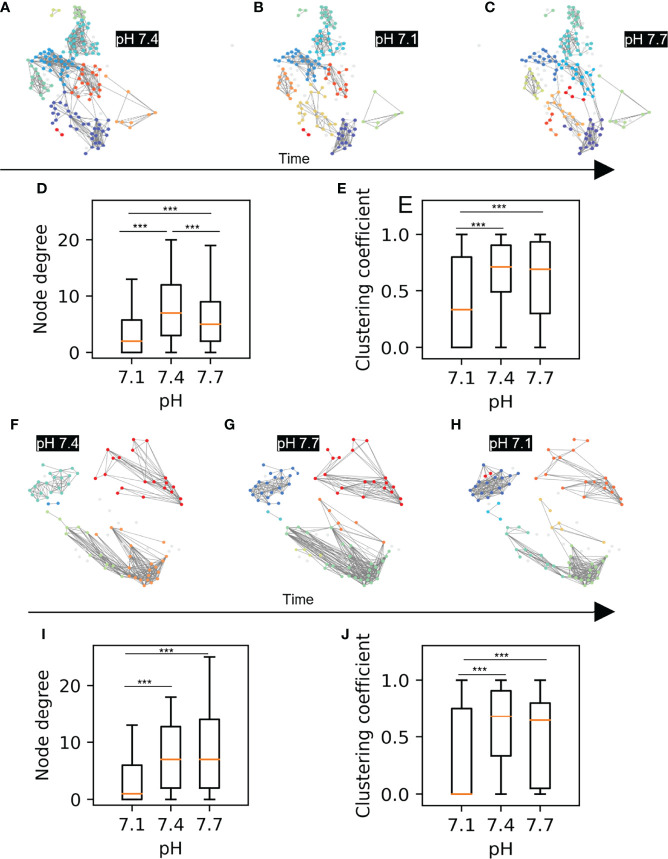
Beta cell functional connectivity at different pH. **(A)** Functional networks of beta cells at 8 mM glucose and different pH changing in a sequence as indicated by the black arrow below: **(A)** 7.4, **(B)** 7.1, **(C)** 7.7. **(D)** Node degree of the pooled data for the sequence A-C. **(E)** Average clustering coefficient of the pooled data for the sequence A-C **(F)** Functional networks of beta cells at 8 mM glucose and different pH changing in a sequence as indicated by the black arrow: **(F)** 7.4, **(G)** 7.7, **(H)** 7.1 **(I)** Node degree of the pooled data for the sequence F-H. **(J)** Average clustering coefficient of the pooled data for the sequence F-H n=5 islets for each condition in the sequence A-C and n=3 for the sequence F-H. Box plots are showing upper and lower quartile; line median; whiskers 5-95%. (***p < 0.001).

Next, we checked whether changes in extracellular pH affect the functional network properties, as has been shown to be the case with glucose stimulation, where an increase in stimulatory glucose results in a greater coordination of the cellular activity within an islet (31). The most glucose-sensitive network parameters established in our previous work were the average node degree and the average clustering coefficient. (20), which indicate the overall coherence and the local synchronicity of [Ca^2+^]_c_ activity, respectively. Using the approach described in Markovič et al. (20), we constructed functional networks of beta cells from recordings for both sequence 1 and sequence 2. In **Figures 3A–C, F–H** we show representative networks extracted at different pH levels, separately for both sequences. Interestingly, despite the fact that under more acidic conditions there is a greater overall activity of beta cells, the underlying functional networks are sparser and more segregated. This observation is quantitatively evidenced by a lower average node degree and a reduced average clustering coefficient (**Figures 3D, E, I, J**). Notably, the network analysis of both sequence protocols showed that in comparison to acidification, alkalinization had a smaller effect on network parameters and can be still dominated by the prior exposure to pH 7.1 (**Figure 3D**).

**Figure 4:**
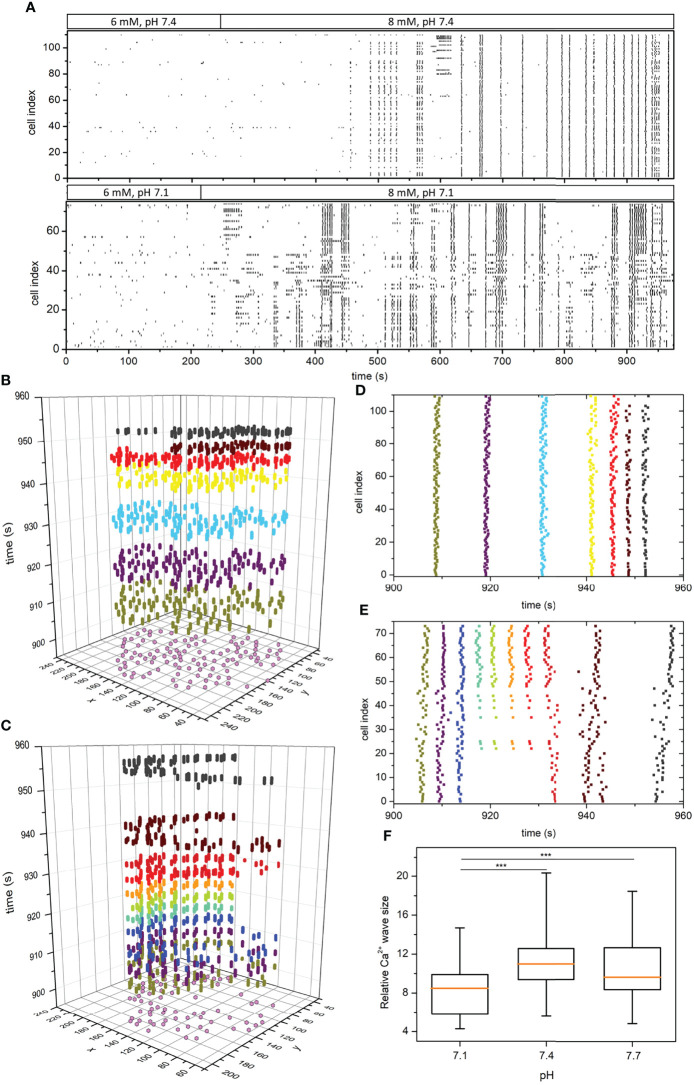
pH-dependent spatiotemporal organization of intercellular Ca^2+^ waves in islets. Raster plots of binarized fast [Ca^2+^]_c_ oscillations of all cells in two representative islets stimulated with 8 mM glucose under pH 7.4 and 7.1, as indicated by the protocol bars **(A)**. Space-time graphs **(B, C)** and the corresponding raster plot outtakes **(D, E)** in the plateau phase of sustained activity under 8 mM glucose and under pH 7.4 **(B, D)** and pH 7.1 **(C, E)**. The colors of dots in panels **(B, C)** signify different extracted Ca^2+^ waves and purple dots in the x-y plane denote the coordinates of individual beta cells. The box-plots in panel **(F)** signify the distribution of relative Ca^2+^ wave sizes under different pH pooled from 3 different islets for each pH level. Box plots are showing upper and lower quartile; line median; whiskers 5-95%. (***p < 0.001).

To investigate the principles of pH-dependent functional connectivity patterns in more detail, we additionally investigated the spatiotemporal organization of intercellular Ca^2+^ waves. We extracted and binarized the fast [Ca^2+^]_c_ oscillations, identified individual waves, and computed their sizes, as described in Materials and methods. In **Figure 4A** we show two typical recordings performed under pH 7.1 and 7.4 in the form of raster plots of binarized calcium activity of all cells within the islets. Additionally, in **Figures 4B–E** we visualize the spatiotemporal behavior for shorter intervals as space-time plots and the corresponding raster plots, in which individual Ca^2+^ waves are indicated by different colours. Evidently, while in pH 7.4 the plateau phase is dominated by global and very coherent Ca^2+^ waves, in pH 7.1 the intercellular activity patterns appear more heterogeneous and less coherent. To put it differently, in acidic conditions we can often observe waves that encompass only a part of the islet, but these waves occur more repeatedly, giving thereby rise to a higher average activity. This is additionally confirmed by the quantification of pooled data (**Figure 4F**), where the average size of Ca^2+^ waves was found significantly smaller under pH 7.1 than under 7.4 or 7.7. We argue that this more erratic nature of spatiotemporal activity leads to decreased compactness of the functional beta cell networks under acidification.

## Discussion

The major contribution of the work presented here is the demonstration that the glucose-induced [Ca^2+^]_c_ oscillations in beta cell collectives depend on extracellular pH. The concentrations of both [H^+^] (20-80 nM, equivalent to pH 7.1-7.7, respectively) and glucose (6-8 mM) were kept in the physiological range. Changes in this pH range are possible *in situ* conditions due to close proximity to activated neighboring ductal and exocrine cells during the process of digestion. Utilizing the advances in both microscopy and data analysis alongside acute pancreatic tissue slice preparation, we were able to perform high precision [Ca^2+^]_c_ measurements (18, 19, 22). This enabled us to assess and describe the function of the individual beta cell and the function of beta cell collectives within the pancreatic islet.

Earlier work has shown that the activation at physiological glucose concentration can be characterized as both synchronous and variable among cells (24, 32–36). This study and one of our previous reports (31) showed a high variability between the activation times of individual cells within the islet during stimulation with physiological glucose concentration. The idea of heterogeneity among beta cells is not new and it has been proposed decades ago (37). Only recent experiments started to accumulate data supporting this claim. The heterogeneity among beta cells is observed on multiple levels, among others: morphology (38, 39), gene and protein expression (40, 41), cell connectivity (42, 43) and functional heterogeneity (44, 45). The heterogeneity we and Stožer et al. (2021) observed on the level of activation time remained preserved at higher than physiological [H^+^]. Elevated [H^+^] induced a clear left shift of the activation curve of beta cells, as clusters representing ~10% of the beta cell population were prematurely activated at this high [H^+^] already in non-stimulatory glucose conditions. Earlier activation can be associated with the ability of H^+^ ions to modulate beta cell activity by directly modulating proteins on the beta cell plasma membrane. The pancreatic-specific pH-gated potassium channel TALK1 exhibits reduced opening probability by increased extracellular [H^+^] (8). OGR1 is another example of membrane protein in beta cells whose function can be modulated by pH (12, 13). Interestingly OGR1 knockout has reduced pH influence on insulin secretion, but the observed enhancement by pH suggesting that additional mechanisms might be involved (13). Future work on beta cells with specific protein ablation or the use of target specific inhibitors of the membrane proteins is needed to dissect these mechanisms.

At the plateau phase, beta cells exhibit highly coordinated activity (46) and at this stage, we regard a mouse islet, during a constant glucose stimulation, functions as a functional syncytium. The plateau phase therefore allows us to investigate parameters such as frequency of [Ca^2+^]_c_ oscillations and the average beta cell activity in an islet. To our knowledge, until now the influence of pH on beta cell [Ca^2+^]_c_ oscillations has not been studied, as the previous studies only demonstrated the pH effects on the electrical activity or insulin secretion (5, 47, 48). The measurements of [Ca^2+^]_c_ oscillations provide insights both into the mechanisms downstream of the electrical activity (49–51), as well as mechanisms triggering insulin secretion (52). Our results upgrade these previous electrophysiological studies and insulin measurements by a high spatiotemporal resolved Ca^2+^ imaging.

To avoid the dilemma whether the pH effects involve extra- or intracellular mechanisms, we focused on its effects on the intercellular communication. Advanced network analysis based on threshold pairwise correlations of Ca^2+^ imaging signals has proven itself a sensitive tool to quantify the non-trivial intercellular interaction patterns in multicellular systems (53, 54), including the islets of Langerhans (17, 20, 24–26, 55). Reaching the glucose concentrations above the physiological threshold for activation of beta cells in mouse islets (6.5-7 mM), it takes about ten minutes of constantly elevated glucose concentration until all small clusters of beta cells distributed all over the islet eventually get recruited into an active collective with a “wave-like” propagation pattern (19, 23, 25, 29, 46, 56). Along with prolonged [Ca^2+^] oscillations and higher activity, progressively higher stimulatory glucose concentration leads to a higher average node degree and clustering coefficient, indicating the network expansion (20, 31). Interestingly, pH 7.1 triggered even higher activity and on average increased the frequency of [Ca^2+^]_c_ oscillations, but the cell network became more segmented compared to pH 7.4 at the same stimulatory glucose concentration. A Ca^2+^ wave analysis has revealed that a decreased compactness of the functional beta cell network structure in acidic conditions is a consequence of a less coherent spatiotemporal activity and more heterogeneous and smaller Ca^2+^ wave sizes. The presence of less connected beta cells and smaller beta cell clusters raised a question about whether larger active clusters and predominantly global Ca^2+^ waves are needed for the normal and more efficient islet function, or it is more beneficial for the beta cell collective sensing if they communicate in near proximity. The reduced gap-junctional coupling could release the break on the most active beta cells by uncoupling their activity from the rest of the syncytium, allowing these cells to activate at a usually substimulatory glucose concentration (57, 58). Furthermore, H^+^ ions can weaken the cellular connections by decreasing gap junction protein expression (59) and by reducing the gap junction coupling efficiency (60). Further studies are needed to determine whether the altered intercellular communication in lower pH is due to the effects on reduced gap junction conductance or increased input resistance of beta cells.

A prolonged increase in [H^+^] is in no way beneficial for the overall health of an organ. The pancreas contains cell types that can control the pH of their microenvironment. Both acinar cells and ductal cells can sense and regulate the pH. It might be that under physiological conditions, an initial increase in [H^+^] from a local source, like a neighboring ductal cell is sufficient to enhance the response to increased glucose concentration, while prolonged acidic conditions are less probable due to mechanisms of local pH control. The experimental model used in this study could be used to mimic the cellular responses during the early disease progression such as pancreatitis (61–63).

In the scope of this paper, we confirmed previous findings that the pancreatic beta cell activities can be modulated by the extracellular pH. Moreover, we obtained the data using mouse acute pancreatic tissue slice with preserved tissue architecture of the islets alongside the surrounding cells. The [Ca^2+^]_c_ oscillations were triggered within the physiological range of both glucose and pH. This approach enabled us to study individual beta cells as well as beta-cell collective responses, revealing until now uninvestigated pH-dependent network properties within pancreatic islets. The data clearly demonstrate that under these conditions decreasing pH enhanced beta cell activity and insulin secretion as well as it altered the nature of intercellular communication patterns.

## Data Availability Statement

The raw data supporting the conclusions of this article will be made available by the authors, without undue reservation.

## Ethics Statement

The animal study was reviewed and approved by Federal Ministry of Education, Science and Research of the Republic of Austria (permit number: 2020-0.258.669). Veterinary Administration of the Republic of Slovenia (permit number: U34401-35/2018-2).

## Author Contributions

Conceptualization, SP, W-HT, MG, S-BY, and MS; methodology, SP, MG, SS, AS, and DK; writing-original draft preparation, SP and MS; writing-review and editing, SP, JP, SS, MG, W-HT, AS, DK, S-BY, and MS; funding acquisition, S-BY and MS. All authors have read and agreed to the published version of the manuscript.

## Funding

MS receives grants by the Austrian Science Fund/Fonds zur Förderung der Wissenschaftlichen Forschung (bilateral grants I3562-B27 and I4319-B30) and National Institutes of Health (1R01DK127236). MS and AS further received financial support from the Slovenian Research Agency (research core funding programs P3-0396 and I0-0029, as well as projects N3-0048, N3-0133, J3-3077, and J3-9289). S-BY is supported by the Institute of Biomedical Sciences at Academia Sinica and the Ministry of Science and Technology (106-2320-B-001-013, 107-2320-B-001-026-MY3 and 110-2314-B-001 -007).

## Conflict of Interest

The authors declare that the research was conducted in the absence of any commercial or financial relationships that could be construed as a potential conflict of interest.

## Publisher’s Note

All claims expressed in this article are solely those of the authors and do not necessarily represent those of their affiliated organizations, or those of the publisher, the editors and the reviewers. Any product that may be evaluated in this article, or claim that may be made by its manufacturer, is not guaranteed or endorsed by the publisher.
